# Effect of electroacupuncture on the repair of stress ulcer injury in neurocritical patients: A randomized clinical trial

**DOI:** 10.3389/fmed.2022.1001584

**Published:** 2022-11-16

**Authors:** He Li, Li-li Li, Jian Wang, Yong-qiang Wang, Lan Wang, Lan Yuan, Wen-ting Chen, Jian-gang Song

**Affiliations:** ^1^Department of Anesthesiology, Shuguang Hospital Affiliated to Shanghai University of Traditional Chinese Medicine, Shanghai, China; ^2^Acupuncture and Anesthesia Research Institute, Shuguang Hospital Affiliated to Shanghai University of Traditional Chinese Medicine, Shanghai, China

**Keywords:** stress ulcer, severe neurological disease, electroacupuncture, gastric mucosal injury, repair factors

## Abstract

**Background:**

Stress ulcer (SU) is one of the main causes of prolonged hospital stay, poor prognosis, and increased mortality in critically ill patients. This study aimed to investigate the effect of electroacupuncture (EA) on SU in patients with severe neurological diseases and explore its possible mechanisms.

**Methods:**

Taking patients with SU in adult neurocritical care as the research object, they were randomly divided into the EA group and the control group. Through the perioperative EA intervention, the following indicators were documented: main observation indicator (the effective rate of SU treatment), secondary observation indicators (gastric juice pH, gastric juice occult blood test, and stool occult blood test), related mechanisms [repair factors trefoil factor family 2 (TFF2), vascular endothelial growth factor (VEGF), and heat shock protein 70 (HSP70)], complications during hospitalization, and intensive care unit (ICU) stay time.

**Results:**

Compared with the control treatment, EA increased the effective rate of SU treatment (85.4% for the EA group, 57.5% for the control group, risk difference: 27.9% (95% CI: 8.3%–45.1%); *P* < 0.01). EA increased the success rate of gastric juice pH treatment on days 1, 2, and 3 (*P* < 0.01 for day 1, *P* < 0.05 for days 2 and 3). EA lowered the positive rate of gastric occult blood test on days 1 and 3 (all *P*-values < 0.05) and the positive rate of fecal occult blood test on day 3 (*P* < 0.05). EA also reduced the ICU stay time (*P* < 0.01) and total hospitalization time (*P* < 0.05). Compared with day 0, all serum repair factors (VEGF, HSP70, and TFF2) of both groups significantly increased on days 1, 3, and 5 (all *P*-values < 0.01). Compared with the control group, VEGF in the EA group was increased on days 3 and 5 (all *P*-values < 0.01); HSP70 was increased on days 1, 3, and 5 (*P* < 0.05 for day 1, *P* < 0.01 for days 3 and 5); and TFF2 was increased on days 1, 3, and 5 (all *P*-values < 0.01).

**Conclusion:**

Electroacupuncture promoted the repair of SU damage in severe neurological disease, and its effect was related to enhancing the expression of gastric mucosal repair factors.

**Clinical trial registration:**

[https://www.chictr.org.cn/showprojen.aspx?proj=127012], identifier [ChiCTR2100046701].

## Introduction

The incidence of stress ulcer (SU) is approximately 70–90% in critically ill patients (1), whose injury is characterized by multiple scattered erosions in the gastric mucosa and/or hemorrhage in superficial areas (1). Hemorrhage, occurring mostly from capillaries on the surface of the gastric mucosa, is diffuse and, therefore, difficult to control. Over the past 20 years, SU and its associated bleeding have been the leading cause of prolonged hospitalization, poor prognosis, and increased mortality in critically ill patients (2–4).

Currently, the main clinical measures to prevent and treat SU include enteral nutrition, analgesia, sedation, thromboembolism prevention, and blood glucose control (5). Pharmacotherapy focuses on prevention, including H2 receptor blockers (H2RAs), sucralfate, and proton pump inhibitors (PPIs) (6, 7). However, unfortunately, recent studies have shown that these treatments do not significantly improve the prognosis of critically ill patients; in contrast, they also induce an increased incidence of gastrointestinal bacterial overgrowth, impaired white blood cell function, and hospital-acquired pulmonary infections, resulting in a significant increase in the length of hospital stay associated with non-disease factors in critically ill patients (8–10). Thus, it is an urgent clinical problem to evaluate the risk/benefit for critically ill patients, explore safer, more effective, and easily accepted intervention methods to treat SU, and promote the rapid recovery of critically ill patients.

Acupuncture or electroacupuncture (EA), as an effective treatment in traditional Chinese medicine, is widely used in the prevention and treatment of various clinical diseases. Acupuncture or EA is also commonly used to treat gastrointestinal diseases and has a significant effect on regulating gastrointestinal function, promoting recovery of gastric motility, and treating gastrointestinal injury (11–13). Recent animal experiments have also confirmed that EA at “ST36 (Zusanli) point” can inhibit SU induced by ethanol or non-steroidal anti-inflammatory drugs in rats, increase gastric mucosal blood flow, and promote the repair of the gastric mucosa (14, 15). Both clinical and basic studies have shown that EA can treat SU. However, the effect of EA treatment on SU in neurocritical patients has not been explored, and there is also a lack of high-quality clinical trials. Our group performed a randomized, controlled clinical trial to determine the clinical efficacy of EA on neurocritical patients complicated with SU and evaluate whether this clinical efficacy is related to promoting the release of related repair factors.

## Materials and methods

### Study design and participants

This study was approved by the Institutional Review Board of Shuguang Hospital Affiliated with the Shanghai University of Traditional Chinese Medicine (2020-sgys-050). Representatives of patients signed the informed consent form registered in the Chinese Clinical Trial Registry (ChiCTR2100046701).

Participants aged 18–75 years with body mass index (BMI) of 18–30 kg/m^2^ and American Society of Anesthesiologists (ASA) physical status class of ≤ III, and those scheduled for emergency decompressive craniectomy and intracranial hematoma removal, combined with SU, were eligible for trial inclusion.

Diagnosis of SU was based on preoperative drainage of bloody or coffee-colored fluid from the gastric tube and a positive occult blood test of the drainage fluid.

Exclusion criteria included heart failure, severe respiratory insufficiency, liver and kidney dysfunction, blood system diseases, mental illness, receiving hormonal medication within 7 days prior to surgery, the use of aspirin and other anticoagulant drug therapies, pregnancy or lactation, local skin infection at meridians, allergy, and acupuncture treatment refusal.

### Patients’ randomization and interventions

The patients were divided into two groups by using the Statistical Product and Service Solutions (SPSS) software for block randomization in a 1:1 ratio. This random method ensured that the number of patients in the two groups would be essentially equal with highly efficient enrollment. Study-group assignments were concealed in opaque envelopes and revealed by a research nurse upon patients’ arrival at the operation room. The researchers, including the statisticians, research coordinators, data collectors, and outcome assessors, were blinded to treatment allocation. The care team (surgeons, anesthesiologists, and nurses) were aware that an acupuncture study was undergoing but were blinded to the study hypothesis and intervention protocol. The acupuncturists were not blinded, but they were not involved in the outcome assessment or data analysis. The participants were in a comatose state, and we were unsure whether they were blind to the group assignment.

The patients were divided into two groups, namely, the EA group and the control (C) group. Both groups were given the symptomatic treatment plan based on SU, with reference to the recommendations of experts in the prevention and treatment of SUs in the adult intensive care unit (16, 17). The C group received general anesthesia with endotracheal intubation alone without any acupoint stimulation. The EA group treatment was based on that of the C group, with EA intervention once before surgery. In addition, EA was performed throughout the surgery operation and twice a day for 1–5 days after surgery, once in the morning and once in the afternoon, each time for 30 min (18, 19). The following acupoints were selected: ST36 (Zusanli) and ST34 (Liangqiu) points, of which ST36 points are Foot-Yangming Stomach Meridian and Stomach Xiahe points located in the anterolateral leg three in below ST35 (Dubi) point, a transverse finger (middle finger) from the anterior edge of the tibia. ST34 is also the Foot-Yangming Stomach Meridian point located two in above the lateral edge of the patella between the rectus femoris and vastus lateralis muscle in a straight line with the lateral knee acupoints.

The acupuncturist stabbed the needle vertically into the skin for about 1–1.5 in. The speed of needle advancement was uniform and slow, with consistent twisting and lifting angles and small amplitude. When the acupuncturist felt a sinking sensation under the skin, it meant the needle was applied successfully (we called it “Deqi” in Traditional Chinese Medicine). Later, these points were connected to the EA apparatus *via* electrode wires at the end of the EA needle, ST36 connected to the positive pole and ST34 connected to the negative pole, which were positioned in strict accordance with the National Standard GB 12346-90 of the People’s Republic of China (20). EA parameter was selected with a frequency of 2/100 Hz, having dense waves in alternate position. The current intensity was set to 2 mA.

### Anesthesia and surgery

After admission, the patients received decompressive craniectomy and evacuation of an intracranial hematoma under general anesthesia in time. Gastrointestinal decompression and gastric juice monitoring were routinely performed with an indwelling gastric tube before the operation. After entering the operating room, the patients were monitored for vital signs using a multifunctional parameter monitor. The monitoring contents included heart rate, heart rhythm, invasive/blood pressure, pulse oxygen saturation, nasopharyngeal temperature, and depth of sedation. The central venous puncture set was used for deep venous (internal jugular, subclavian, and femoral) puncture and catheterization, and if necessary, invasive arterial (radial and dorsalis pedis) puncture and catheterization were used to monitor invasive blood pressure. Fluid supplementation was given before anesthesia, and volume prefilling was performed at a crystalloid: colloid ratio of 2:1. The intraoperative fluid therapy protocol referred to the “Expert Consensus on Fluid Therapy during Anesthesia and Surgery (2017).”

Anesthesia was induced with mask oxygen inhalation at 2–4 L/min, intravenous continuous pump injection of dexmedetomidine at 0.3–0.5 μg/kg/h, sufentanil at 0.1–0.2 μg/kg, propofol at 2.0–2.5 mg/kg, and atracurium at 0.15 mg/kg. Muscle relaxation monitoring [train-of-four (TOF) monitoring] was used, and when the TOF was 0 and the bispectral index (BIS) value was between 40 and 50, endotracheal intubation was performed under a video laryngoscope. In addition, the catheter was fixed after the inflation of the catheter balloon, an anesthesia machine was externally connected, and the appropriate ventilation mode (volume control or pressure control) was selected for assisted ventilation.

Anesthesia was maintained with continuous pumping of propofol target-controlled infusion (TCI), with pumping plasma concentration set at 1.5–4 μg/ml. Continuous pumping of remifentanil TCI was given, with pumping plasma concentration set at 1.5–3.5 μg/ml. Additional 0.05–0.1 μg/kg of sufentanil and 1/3–1/5 of the initial dose of atracurium were given as necessary. Sevoflurane was inhaled throughout, and the effective concentration of inhalation was maintained at 0.5–0.7 minimum alveolar concentration (MAC). Intraoperative circulatory fluctuations were maintained at ±20% preoperatively, and the sedation index was maintained between 40 and 60. Adequate oxygenation, adequate cerebral perfusion, and maintenance of the necessary state of cerebral relaxation were also ensured. We avoided hypercapnia, too shallow anesthesia, excessive stress, and severe circulatory fluctuations and maintained acid-base balance and water and electrolyte stability in the internal environment.

Inhalation of anesthetic drugs was stopped 20 min before the end of the surgery, and intravenous administration was stopped 10 min before the end of the surgery. Before skin suturing, 0.375% ropivacaine hydrochloride was given for subcutaneous infiltration anesthesia to play a role in postoperative multimodal analgesia. At the end of the operation, the patient’s vital signs were checked for stability, whether the skin and soft tissue were compressed, and whether the endotracheal tube was displaced. After confirmation, it was sent back to the neurosurgical intensive care unit with the tube.

### Measures

The general patients’ data (e.g., age, gender, BMI, ASA grade, coexisting diseases, type of surgery, the dosage of anesthetic drugs, operation time, and duration of hypotension) were recorded. The main evaluation indicators were the effective rate of postoperative SU treatment, which was measured once every 24 h within 72 h after operation: (1) 48-h effective rate: the characteristics of gastric juice and stool basically returned to normal, the symptoms of gastrointestinal bleeding stopped within 48 h after treatment, the occult blood test of gastric juice was negative, and the vital signs were stable; (2) 72-h effective rate: the symptoms of gastrointestinal bleeding stopped 48–72 h after treatment, and other symptoms were significantly improved; and (3) ineffective: the symptoms of gastrointestinal bleeding did not stop more than 72 h after treatment, and other symptoms were not significantly improved. The effective rate was calculated as the sum of the 48-h effective rate and 72-h effective rate. Secondary evaluation indicators included clinical efficacy indicators (gastric pH, gastric occult blood test, and fecal occult blood test), five gastric pH values were measured within every 24 h, and one gastric occult blood test and one fecal occult blood test were measured within every 24 h for 1–3 days after surgery. For mechanism-related indicators (repair factors TFF2, VEGF, and HSP70), peripheral blood was collected and measured by enzyme-linked immunosorbent assay (ELISA) before 1, 3, and 5 days after surgery. EA-related adverse events were recorded.

Any adverse events related to acupuncture treatment, such as adverse or unanticipated signs, symptoms, or diseases, were reported by acupuncturists and patient representatives. Serious adverse events had to be reported to the principal investigator and the data and safety monitoring committee within 24 h of occurrence.

### Sample size calculation

For power calculation, we estimated the sample size using the effective rate of postoperative SU as the main outcome measure (the main evaluation indicator). According to the results of the preliminary experiment, of the 11 patients in the EA group, EA was significantly effective, effective, and ineffective within 72 h in one, seven, and three cases, respectively, with an overall response rate of 72.7% (8/11). Of the nine patients in the control group, EA was significantly effective, effective, and ineffective within 72 h in no, four, and five cases, respectively, with an overall response rate of 44.4% (4/9). Taking the test level α = 0.05 and the test power 1-β = 0.8 into account, the calculation results were as follows: 36 patients were needed for each group. According to the dropout rate of 10%, 40 patients were needed for each group, with a total of 80 patients needed.

### Statistical analysis

We used the Shapiro–Wilk test and Q-Q plots to test each continuous variable for positivity. Continuous variables obeying normal distribution were expressed as mean ± standard deviation (SD). Continuous variables not obeying normal distribution were expressed as median interquartile range (IQR), and categorical variables were expressed as number and percentage. For non-normal continuous and categorical variables, we used the Mann–Whitney *U* test and Fisher’s exact test to test for differences between the two groups. For variables regarding demographics, baseline characteristics, and surgical characteristics that obeyed a normal distribution, we used Student’s *t* test for differential analysis between the two groups. In addition, we used an analysis of covariance (ANCOVA) for neurological assessment and mechanical ventilation for differential analysis. For repeated measures data, we analyzed differences between the groups by repeated measures ANCOVA. All tests were two-sided with a significance level of α = 0.05. Statistical analysis was performed by SPSS program version 21.0 (SPSS Inc., Chicago, IL, USA).

## Results

### Demographic data

**FIGURE 1:**
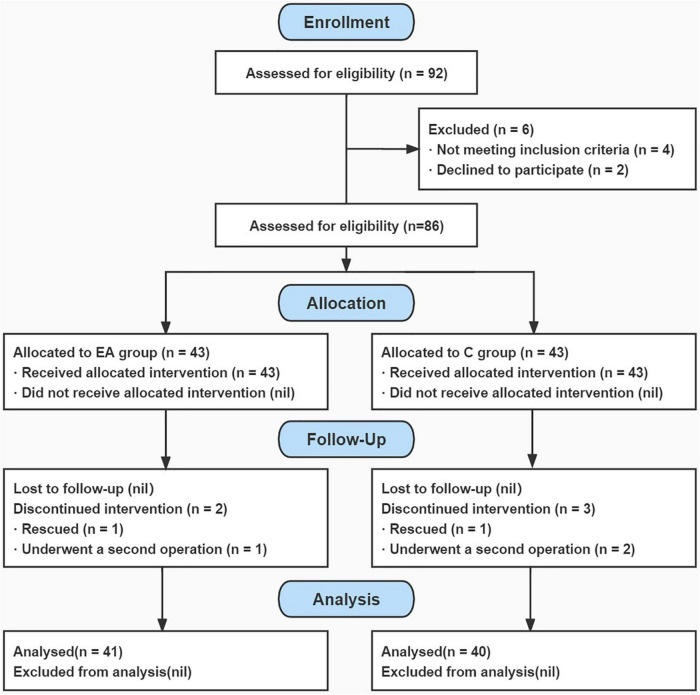
Flowchart of the study process. EA, electroacupuncture; C, control.

A total of 92 neurocritical patients with SU were screened between 1 June 2021, and 30 May 2022. Among them, 86 eligible patients were enrolled and randomly assigned to either the EA group (*n* = 43) or the C group (*n* = 43). A total of 81 (94.2%) patients completed the trial by the time of discharge, and five (5.8%) patients were dropped out of the study owing to intraoperative rescue or emergency reoperation (two in the EA group and three in the C group) (Figure 1 for a flowchart). There were 41 patients in the EA group, including 27 men and 14 women, with a mean age of 52.95 ± 9.79 years (range, 28–71). There were 40 patients in the C group, including 21 men and 19 women, with a mean age of 51.80 ± 11.66 years (range, 27–70 years).

It can be seen from Table 1 that the difference in the baseline data between the two groups had no statistical significance (*P* > 0.05).

**TABLE 1:** Comparison of baseline data between the two groups (EA group *n* = 41, C group *n* = 40, *n*% or *X* ± SD).

	EA group	C group	*P*
Age (y)	52.95 ± 9.79	51.80 ± 11.66	0.950
Gender (M/F)	27/14	21/19	0.159
BMI	28.07 ± 4.54	29.15 ± 5.43	0.336
ASA (II/III)	12/29	18/22	0.108
Comorbid diabetes mellitus (present/absent)	27/14	22/18	0.220
Hemorrhage location (cerebrum/thalamus/cerebellum)	22/13/6	23/13/4	0.814
Bleeding volume (ml)	275.90 ± 110.80	261.23 ± 108.16	0.548
GCS score (mild/moderate/severe/very severe)	0/19/21/1	0/16/22/2	0.738
Type of procedure (drilling/flap removal)	7/34	7/33	0.595
Sufentanil (μg)	30.61 ± 6.54	28.63 ± 4.93	0.128
Atracurium (mg)	16.68 ± 3.45	15.75 ± 4.35	0.289
Propofol (mg)	405.20 ± 186.63	490.53 ± 222.49	0.065
Operative time (min)	151.59 ± 66.55	179.50 ± 81.72	0.095
Hypotension time (min)	15.56 ± 6.61	13.58 ± 5.24	0.139
Dexmedetomidine (μg)	103.29 ± 36.31	94.38 ± 39.24	0.291
Mechanical ventilation time (h)	62.22 ± 21.01	55.40 ± 20.10	0.140

Type of surgery: trepanation = single/trepanation drainage; bone flap removal = bone plate decompression + evacuation of intracranial hematoma, *P* < 0.05, *P* < 0.01.

### Primary outcome

As shown in Table 2, the effective rate of SU treatment in the EA group was significantly increased compared with the C group (*P* < 0.01). In the C group, the 48-h effective rate was 12.5%, the 72-h effective rate was 45%, and the effective rate was 57.5%. After perioperative active symptomatic treatment, 17 patients still had unrelieved gastrointestinal bleeding symptoms after 72 h of treatment. In the EA group, the 48-h effective rate was 26.8%, the 72-h effective rate was 58.5%, and the effective rate was 85.4%. The risk difference in effective rate between the groups was 27.9% (95% CI: 8.3–45.1%). After active treatment, only six patients had gastrointestinal bleeding symptoms after 72 h.

**TABLE 2:** Comparison of clinical efficacy of stress ulcer (SU) treatment between the two groups [(n) %].

Group	Number of subjects	48 h-effective	72 h-effective	Ineffective	Effective rate
EA Group	41	11 (26.8)	24 (58.5)	6 (14.6)	35 (85.4%)
C group	40	5 (12.5)	18 (45)	17 (42.5)	23 (57.5%)
*P*					0.005

Referring to the “Guidelines for the Diagnosis and Treatment of Acute Non-variceal Upper Gastrointestinal Bleeding 2015” to evaluate the efficacy. (1) 48-h effective rate: the characteristics of gastric juice and stool basically returned to normal, the symptoms of gastrointestinal bleeding stopped within 48 h after treatment, the occult blood test of gastric juice was negative, and the vital signs were stable. (2) 72-h effective rate: the symptoms of gastrointestinal bleeding stopped 48–72 h after treatment, and other symptoms were significantly improved. (3) Ineffective: the symptoms of gastrointestinal bleeding did not stop more than 72 h after treatment, and other symptoms were not significantly improved. The overall response rate was the sum of the 48-h effective rate and 72-h effective rate (43).

### Secondary outcome

**FIGURE 2:**
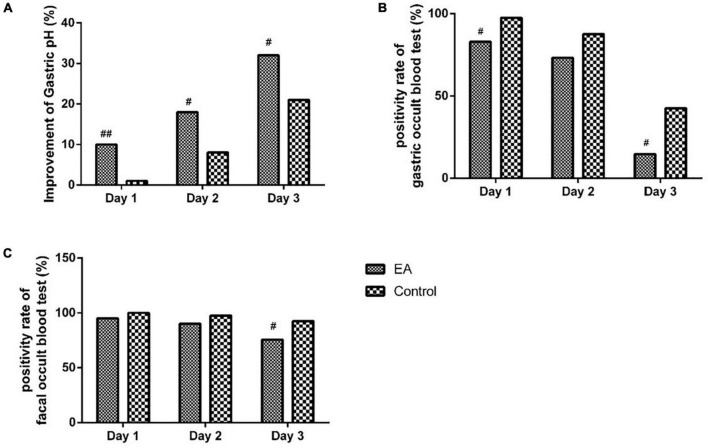
Comparison of gastric pH, occult gastric blood, and fecal occult blood test within 3 days after operation between the two groups. **(A)** Comparison of gastric pH within 3 days after operation between the EA and control groups. **(B)** Comparison of gastric occult blood test within 3 days after operation between the EA and control groups. **(C)** Comparison of fecal occult blood test within 3 days after operation between the EA and control groups. Day 1 = 1 day after operation; day 2 = 2 days after operation; day 3 = 3 days after operation. ^#^*P* < 0.05, ^##^*P* < 0.01.

As shown in Figure 2, compared with the C group, the treatment success rate of gastric pH in the EA group was significantly increased on days 1 to 3 (*P* < 0.01 for day 1, *P* < 0.05 for days 2 and 3). Compared with the C group, the positive rate of gastric occult blood test in the EA group was significantly decreased on days 1 and 3 (all *P*-values < 0.05). There was no significant difference in the positive rate of gastric occult blood test between the two groups on day 2 (*P* > 0.05). Compared with the C group, the positive rate of fecal occult blood test in the EA group was significantly decreased on day 3 (*P* < 0.05). There was no significant difference in fecal occult blood test between the two groups on days 1 and 2 (*P* > 0.05).

**FIGURE 3:**
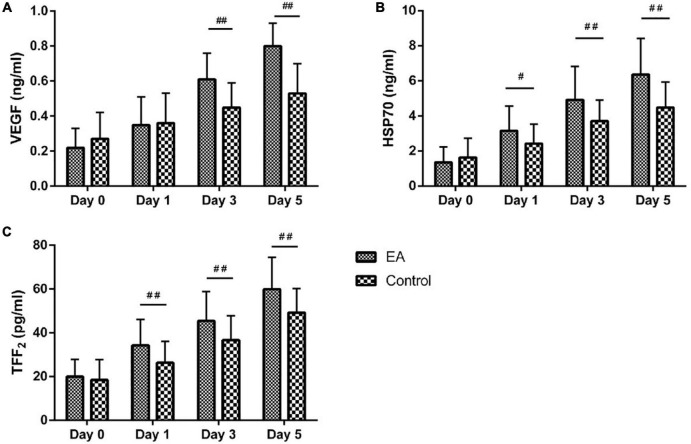
Comparison of serum vascular endothelial growth factor (VEGF), heat shock protein 70 (HSP70), and trefoil factor family 2 (TFF2) levels between the two groups. **(A)** Comparison of serum VEGF level between the EA and control groups. **(B)** Comparison of serum HSP70 level between the EA and control groups. **(C)** Comparison of serum TFF2 level between the EA and control groups. Day 0 = after admission, before operation; day 1 = 1 day after operation; day 3 = 3 days after operation; day 5 = 5 days after operation. ^#^*P* < 0.05, ^##^*P* < 0.01.

The trends of serum repair factors with a time of both groups are plotted in Figure 3. Compared with day 0, all serum repair factors (VEGF, HSP70, and TFF2) of both groups significantly increased at all the subsequent time points (days 1, 3, and 5) with all *p*-values of < 0.01. Compared with the C group, the serum repair factor VEGF of patients in the EA group was significantly increased from day 3 to day 5 (all *P*-values < 0.01). Compared with the C group, the serum repair factor HSP70 of patients in the EA group was significantly increased from day 1 to day 5 (*P* < 0.05 for day 1, *P* < 0.01 for days 3 and 5). Compared with the C group, the serum repair factor TFF2 of patients in the EA group was significantly increased from day 1 to day 5 (all *P*-values < 0.01).

**FIGURE 4:**
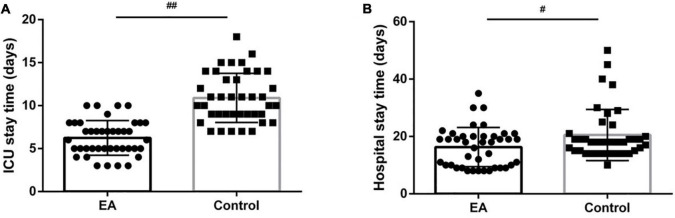
Comparison of postoperative intensive care unit (ICU) stay and hospital stay between the two groups. **(A)** ICU stay; **(B)** hospital stay. ^#^*P* < 0.05, ^##^*P* < 0.01.

Figure 4 shows that the length of ICU stay was significantly reduced in the EA group compared with the C group (*P* < 0.01), and the length of hospital stay was significantly reduced (*P* < 0.05).

## Discussion

Our study found that the EA significantly improved the effective rate of SU treatment in neurocritical patients, including cessation of symptoms of gastrointestinal bleeding and the negative results of the gastric occult blood test. In addition, EA significantly increased VEGF expression in the serum of patients on postoperative days 3 and 5 and significantly increased the expression of HSP70 and TFF2 on postoperative days 1, 3, and 5. Furthermore, the length of ICU stay and hospital stay was significantly reduced. We also found that the effectiveness of EA in promoting repair after SU injury might be associated with elevated expression of repair factors.

Stress-related mucosal disease (SRMD) is a common complication, secondary to active gastrointestinal bleeding caused by SUs, in critically ill patients, causing hemodynamic instability, increasing clinical blood transfusion requirements, and leading to higher hospitalization rates and in-hospital mortality (21–23). Thus, effective treatment of SRMD-related gastrointestinal bleeding has extremely important clinical significance for effectively improving patient prognosis and survival (24–27).

Acupuncture has been used to treat gastrointestinal disorders for many years. Multiple studies have confirmed that EA is effective in the treatment of esophageal motility disorders, chronic atrophic gastritis, and gastric ulcers (28, 29). Zhao et al. (30) have shown the effectiveness of acupuncture in the treatment of SU in patients with stroke. Compared with the control group, the effective rate of treatment in the acupuncture group was significantly higher on post-treatment days 3 and 7. However, such findings were hampered by flaws in the study design. Furthermore, the effect of EA treatment for SUs in neurocritical patients has not been explored. This strictly designed, rigorously conducted trial provided important clinical evidence about the role and value of EA as an effective therapy for treating SUs in neurocritical patients for the first time.

ST36 is an important point of the Foot Yang Ming stomach meridian, which has high efficacy in alleviating stomach pain, vomiting, bloating, diarrhea, constipation, etc. Studies have confirmed that acupuncture at the ST36 has the ability to regulate intestinal peristalsis, increase the activity of various digestive enzymes, and improve blood flow in the gastrointestinal mucosa, which is important, specific, and widespread in the prevention and treatment of gastric diseases (31). Animal experiments have also confirmed the protective effect of acupuncture ST36 on gastric mucosa (32, 33). ST34 is also an acupoint of the Foot Yang Ming stomach meridian, which locates at the end of the extremities and has the interaction of the yin and the yang. ST34 regulates the peristaltic function of the gastrointestinal tract, which has a bidirectional effect on the gastrointestinal environment (34, 35). Therefore, this study has selected ST36 and ST34 as important treatment points to confirm the effectiveness of EA in promoting repair after SU injury.

Previous literature has shown that the mechanism of EA in promoting SU repair mainly included the following four aspects: (1) regulating the secretion of gastrointestinal hormones (e.g., calcitonin, gastrin, and prostaglandins) through central regulation, controlling gastric acid secretion and gastrointestinal motility, and promoting ulcer repair (36, 37); (2) increasing gastric mucosal blood flow by increasing blood perfusion in the ischemic area of the gastric mucosa, meeting the oxygen supply required for local tissue metabolism, reducing the accumulation of metabolites, such as carbon dioxide and hydrogen ions, and preventing further acidification of the gastric mucosa (38); (3) inhibiting inflammatory mediator response, reducing inflammatory injury of gastric mucosa, and promoting repair by inhibiting the release of inflammatory factors, such as interleukin-2, interleukin-6, and tumor necrosis factor-α (39); and (4) promoting the release of repair factors, mainly including the participation of repair factors such as TFF2, VEGF, and HSP70, promoting the regeneration and repair of gastric epithelial cells, and maintaining gastric mucosal barrier integrity (40–42). The results of our study also revealed that the effect of EA in promoting repair after SU injury in neurocritical patients might be related to the elevated expression of repair factors. Whether there are other mechanisms involved in the clinical effects of EA in neurocritical patients is a subject of further studies.

## Limitation

Our study had several limitations. First, acupuncturists were not blinded to the group assignment owing to the treatment procedure. Although the acupuncturists were not involved in the outcome assessment or data analysis, the potential bias would still exist. Second, the patients could not be blinded to the intervention totally as they might sense the acupoint stimuli in the EA group, which might inevitably increase the placebo response (neurocritical patients might have a transient state of wakefulness during the 5 days after surgery). Third, the diagnosis of SU in this study was based on preoperative drainage of bloody or coffee-colored fluid from the gastric tube and a positive occult blood test of the drainage fluid. It is regrettable that we were not able to use a gastroscope to diagnose SU due to the limitations of the critical condition of neurocritical patients, which is an area for improvement in our further clinical studies in the future. In addition, this was a single-center study. While internal validity was not impacted, the external validity and generalizability of the results might have been affected by clinical practice and therapeutic measures in various medical centers. The sample size was relatively small; thus, the power of this intervention should be tested in a clinical trial with a larger sample size.

## Conclusion

This randomized clinical trial found that perioperative EA treatment was an effective therapy for promoting the repair of SU in neurocritical patients, and its effectiveness might be associated with elevated expression of gastric mucosal repair factors. Further studies should focus on a larger sample size and a multicenter randomized clinical trial to verify these findings.

## Data availability statement

The raw data supporting the conclusions of this article will be made available by the authors, without undue reservation.

## Ethics statement

The studies involving human participants were reviewed and approved by the Ethics Committee of Shuguang Hospital Affiliated to Shanghai University of Traditional Chinese Medicine (No. 2020-sgys-050). Representatives of the patients provided their written informed consent to participate in this study.

## Author contributions

W-tC and J-gS: study conception, drafted the manuscript, and obtained funding. JW and Y-qW: acquisition or interpretation of data. L-lL and HL: statistical analyses. HL, W-tC, and J-gS: critically revised the manuscript for important intellectual content. LW and LY: provided administrative, technical, or material support. All authors contributed to the article and approved the submitted version.
